# Allopurinol ameliorates high fructose diet induced hepatic steatosis in diabetic rats through modulation of lipid metabolism, inflammation, and ER stress pathway

**DOI:** 10.1038/s41598-021-88872-7

**Published:** 2021-05-10

**Authors:** In-Jin Cho, Da-Hee Oh, Jin Yoo, You-Cheol Hwang, Kyu Jeung Ahn, Ho-Yeon Chung, Soung Won Jeong, Ju-Young Moon, Sang-Ho Lee, Sung-Jig Lim, In-Kyung Jeong

**Affiliations:** 1 Department of Endocrinology and Metabolism, Kyung Hee University School of Medicine, Kyung Hee University Hospital at Gangdong, #892 Dongnam-ro, Gangdong-gu, Seoul, 05278 Korea; 2 Division of Endocrinology and Metabolism, Department of Internal Medicine, Kyung Hee University School of Medicine, Seoul, Korea; 3 Division of Gastroenterology and Hepatology, Department of Internal Medicine, Soonchunhyang University College of Medicine, Seoul, Korea; 4 Division of Nephrology, Department of Internal Medicine, Kyung Hee University School of Medicine, Seoul, Korea; 5 Department of Pathology, Kyung Hee University School of Medicine, Seoul, Korea

**Keywords:** Metabolic syndrome, Type 2 diabetes, Endocrinology, Pathogenesis

## Abstract

Excess fructose consumption contributes to development obesity, metabolic syndrome, and nonalcoholic fatty liver disease (NAFLD). Uric acid (UA), a metabolite of fructose metabolism, may have a direct role in development of NAFLD, with unclear mechanism. This study aimed to evaluate role of fructose and UA in NAFLD and explore mechanisms of allopurinol (Allo, a UA lowering medication) on NAFLD in Otsuka Long-Evans Tokushima Fatty (OLETF) rats fed a high fructose diet (HFrD), with Long-Evans Tokushima Otsuka (LETO) rats used as a control. There were six groups: LETO, LETO-Allo, OLETF, OLETF-Allo, OLETF-HFrD, and OLETF-HFrD-Allo. HFrD significantly increased body weight, epididymal fat weight, and serum concentrations of UA, cholesterol, triglyceride, HbA1c, hepatic enzymes, HOMA-IR, fasting insulin, and two hour-glucose after intraperitoneal glucose tolerance tests, as well as NAFLD activity score of liver, compared to the OLETF group. Allopurinol treatment significantly reduced hepatic steatosis, epididymal fat, serum UA, HOMA-IR, hepatic enzyme levels, and cholesterol in the OLETF-HFrD-Allo group. Additionally, allopurinol significantly downregulated expression of lipogenic genes, upregulated lipid oxidation genes, downregulated hepatic pro-inflammatory cytokine genes, and decreased ER-stress induced protein expression, in comparison with the OLETF-HFrD group. In conclusion, allopurinol ameliorates HFrD-induced hepatic steatosis through modulation of hepatic lipid metabolism, inflammation, and ER stress pathway. UA may have a direct role in development of fructose-induced hepatic steatosis, and allopurinol could be a candidate for prevention or treatment of NAFLD.

## Introduction

Nonalcoholic fatty liver disease (NAFLD) is a chronic liver disease that occurs in patients with modest or no alcohol use and no viral hepatitis. It is characterized by the accumulation of excess lipids inside hepatocytes^1^. NAFLD ranges from simple steatosis, which is a benign condition, to nonalcoholic steatohepatitis (NASH), which can progress to cirrhosis and hepatic failure^1^. NAFLD usually emerges alongside insulin resistance associated medical conditions (i.e., obesity, type 2 diabetes, and dyslipidemia), and is considered a hepatic manifestation of metabolic syndrome^2^. A dramatic increase in worldwide obesity prevalence has also resulted in increased NAFLD prevalence^3^. NAFLD prevalence rates in Western and Asian countries during the past 20–30 years have been approximately 20–30% and 25% of the general population, respectively^4^. Currently, NAFLD is one of the most common liver diseases, and its clinical burden is expected to rise in the future.

Specific dietary factors, including high levels of saturated fats, cholesterol, or fructose, contribute to the development of obesity, metabolic syndrome, and NAFLD^5^. In particular, consumption of fructose, a dietary monosaccharide, has increased over the last century due to the usage of high-fructose corn syrup as a major sweetener in soft drinks^6^. Two pathogenic mechanisms have been suggested for the HFrD-induced development of NAFLD. First, fructose is lipogenic during its metabolism in the liver^7^. Second, uric acid, a metabolite of fructose metabolism, may have an additional role in the development of fatty liver^8^. Fructose is primarily metabolized in the liver, where it is first phosphorylated by fructokinase. This process requires ATP, which leads to increased intracellular uric acid concentrations, resulting in hyperuricemia^9^. Uric acid generation is a unique feature of fructose metabolism, distinguishing it from glucose metabolism. Previous research has shown that chronic fructose consumption significantly increases both fasting plasma uric acid and 24 h uric acid concentrations in humans^10^. Furthermore, it has been shown in cross-sectional human studies that hyperuricemia is independently associated with hepatic steatosis^11^ and serum uric acid (UA) concentration is an independent risk factor for NAFLD^12^.

Lifestyle modification to reduce body weight is the primary recommended treatment for NAFLD, similar to metabolic syndrome treatment. Weight loss through caloric restriction and exercise is safe and effective in the management of NAFLD, but it is difficult to reach and maintain weight loss target^13^. Several pharmacological agents, including ursodeoxycholic acid^14^, pentoxifylline^15^, statins^16^, thiazolidinediones^17^, and Vitamin E therapy^18^ have been employed in attempts to improve liver histology. Although many drugs are currently being developed and investigated, there are no pharmacological agents approved for NAFLD treatment^19^.

Uric acid may directly affect NAFLD development in HFrD-induced hepatic steatosis, and as such, uric acid level lowering treatment may improve NAFLD. However, the pathogenic molecular mechanism of uric acid in the development of NAFLD has not been completely elucidated.

The Otsuka Long-Evans Tokushima Fatty (OLETF) rat is a well-known animal model of metabolic syndrome, including insulin resistance, obesity, hypertension, dyslipidemia, hyperglycemia, and hepatic steatosis^20^. Hyperuricemia and increased urinary UA excretion have been reported in OLETF rats^21^. However, as hyperglycemia and hyperuricemia in OLETF rats are mild, we treated OLETF rats with a high fructose diet (OLETF-HFrD) to exaggerate hyperuricemia and investigated the effect of allopurinol on metabolic parameters and NAFLD in LETO, OLETF, and OLETF-HFrD rats.

Therefore, this study aimed to evaluate the role of fructose and uric acid on the development of fatty liver disease and explore the effects and mechanisms of allopurinol, a uric acid lowering treatment, on NAFLD using HFrD-fed OLETF rats and OLETF rats.

**Table 1:** Metabolic variables in LETO, OLETF, and HFrD-fed OLETF rats with or without allopurinol treatment after the 16 weeks of study period.

	LETO	LETO-Allo	OLETF	OLETF-Allo	OLETF-HFrD	OLETF-HFrD-Allo
Body weight (g)	545.5 [516.3–570.2]	558.4 [540.5–586.3]	612.0† [607.5–617.9]	610.1† [601.1–615.1]	699.5†* [627.8–737.5]	622.5†^#^ [595.0–647.0]
Pancreas weight (g)	1.0 ± 0.2	1.1 ± 0.3	0.7 ± 0.2	0.9 ± 0.5	0.8 ± 0.3	0.7 ± 0.2
Epididymal fat weight (g)	5.9 ± 1.7	6.0 ± 0.6	5.8 ± 0.6	4.9 ± 1.0	9.0 ± 2.0†*	6.9 ± 1.1^#^
Uric acid, (mg/dL)	0.75 ± 0.1	0.73 ± 0.03	1.60 ± 0.07†	1.33 ± 0.16†*	2.05 ± 0.24†*	1.60 ± 0.13†^#^
Cholesterol, (mg/dL)	94 ± 4	96 ± 5	106.8 ± 6.7†	104.1 ± 10.3†	138.0 ± 16.4†*	91.0 ± 6.2^#^
Triglycerides, (mg/dL)	30 ± 10	28 ± 14	152.8 ± 19.8†	147.3 ± 21.6†	197.8 ± 23.5†*	184.0 ± 11.7†*
HbA1c (%)	3.6 [3.2–4.2]	3.7 [3.3–4.4]	8.25† [7.80–8.70]	8.18† [7.5–8.7]	9.60†* [8.50–10.7]	9.50†* [8.70–10.3]
AST (IU/L)	72.1 ± 15.3	73.1 ± 17.4	123.7 ± 7.2†	121.4 ± 8.1†	118.4 ± 9.0†	143.0 ± 24.4†
ALT (IU/L)	48.1 ± 4.9	43.5 ± 6.8	54.3 ± 7.4†	51.9 ± 9.2	59.8 ± 3.5†*	51.8 ± 4.9#

Tm, temperature; SREBP-1c, sterol regulatory element binding protein 1c; SCD-1, stearoyl-CoA desaturase 1; PPARα, peroxisome proliferator-activated receptor alpha; CPT-1, carnitine palmitoyltransferase 1; TNF-α, tumor necrosis factor alpha; PAI-1, plasminogen activator inhibitor 1.

## Results

### Effects of HFrD and allopurinol treatment on metabolic parameters

Body weight, organ weights, and serum biochemical parameters were measured after the 16-week study period in the LETO, LETO-Allo, OLETF, OLETF-Allo, OLETF-HFrD, and OLETF-HFrD-Allo groups (Table 1). Body weight, as well as serum UA, cholesterol, triglyceride (TG), HbA1C, aspartate aminotransferase (AST), and alanine aminotransferase (ALT) levels, were significantly higher in OLETF rats than in LETO rats (*P* < 0.05). These parameters, in addition to epididymal fat weight, of the OLETF-HFrD group were significantly higher than those of the OLETF group (*P* < 0.05). There was no significant difference in the body weight and serum levels of lipid, HbA1c, and hepatic enzymes between the LETO and LETO-Allo groups. In addition, in the OLETF-Allo group, these parameters were not significantly different from those in the OLETF group, even though the uric acid level of the OLETF-Allo group was significantly lower than that of the OLETF group (*P* < 0.05). However, rats in the OLETF-HFrD-Allo group had significantly lower body weight, epididymal fat weight, and serum levels of UA, cholesterol, TG, and ALT, compared to the OLETF-HFrD group (*P* < 0.05).

**Table 2:** Intraperitoneal glucose tolerance test-derived insulin sensitivity and secretion indices.

	LETO	LETO-Allo	OLETF	OLETF-Allo	OLETF-HFrD	OLETF-HFrD-Allo
**Glucose (mg/dL)**						
0 min	95.6 ± 2.0	97.6 ± 2.0	106.3 ± 5.1	104.0 ± 2.7	115.8 ± 3.6†	110.1 ± 5.3†
30 min	168.3 ± 11.0	165.3 ± 25.8	320.0 ± 31.5†	318.3 ± 31.5†	357.8 ± 46.8†	312.3 ± 54.2†
120 min	154.3 ± 24.8	151.3 ± 26.9	178.0 ± 13.9	177.0 ± 18.0	351.5 ± 37.4†*	337.5 ± 68.0†*
AUC(0–120)	641.7 ± 40.2	632.2 ± 48.5	1093 ± 125†	1080 ± 102.9†	1358 ± 108.8†*	1297 ± 158.2†*
**Insulin (μIU/mL)**						
0 min	37.5 ± 14.5	39.1 ± 10.2	35.3 ± 9.0	32.6 ± 10.3	50.4 ± 6.4†*	50.3 ± 11.6†*
30 min	127.5 ± 56.2	101.9 ± 15.0	78.0 ± 36.1†	67.9 ± 20.3†	99.0 ± 46.6*	110.9 ± 21.5*
120 min	35.5 ± 3.6	15.7 ± 3.3†	51.4 ± 21.4†	42.8 ± 18.8	68.6 ± 27.4†*	57.8 ± 36.1†
AUC (0–120)	326.7 ± 57.6	250.1 ± 19.4†	223.9 ± 29.3†	213.3 ± 33.4†	303.2 ± 38.9*	298.7 ± 39.3*
**IGI 30**	1.18 ± 0.39	1.06 ± 0.37	0.29 ± 0.32†	0.18 ± 0.09†	0.25 ± 0.33†	0.43 ± 0.26†*
**HOMA-beta**	405.7 ± 117.3	407.4 ± 101.2	307.7 ± 98.6†	282.8 ± 61.3†	345.6 ± 27.1*	390.2 ± 112.1*#
**HOMA-IR**	8.9 ± 3.7	9.4 ± 2.6	9.2 ± 2.4	8.4 ± 3.0	14.5 ± 2.6†*	12.8 ± 3.0#

Data are expressed as mean ± SE or median with interquartile range.

LETO, Long-Evans Tohushima Otsuka; LETO-Allo, LETO rats treated with allopurinol; OLETF, Otsuka Long-Evans Tokushima Fatty rats; OLETF-Allo, OLETF rats treated with allopurinol; OLEFT-HFrD, OLETF rats fed with high fructose diet; OLETF-HFrD-Allo rats, OLETF rats fed with high fructose diet which were treated with allopurinol; HFrD, high fructose-diet; HbA1c, hemoglobin A1c; AST, aspartate aminotransferase; ALT, alanine aminotransferase.

^†^*P* < 0.05 versus LETO group, *P < 0.05 versus OLETF group, #*P* < 0.05 versus OLETF-HFrD group.

### Effects of HFrD and allopurinol treatment on glucose metabolism parameters

**Figure 1:**
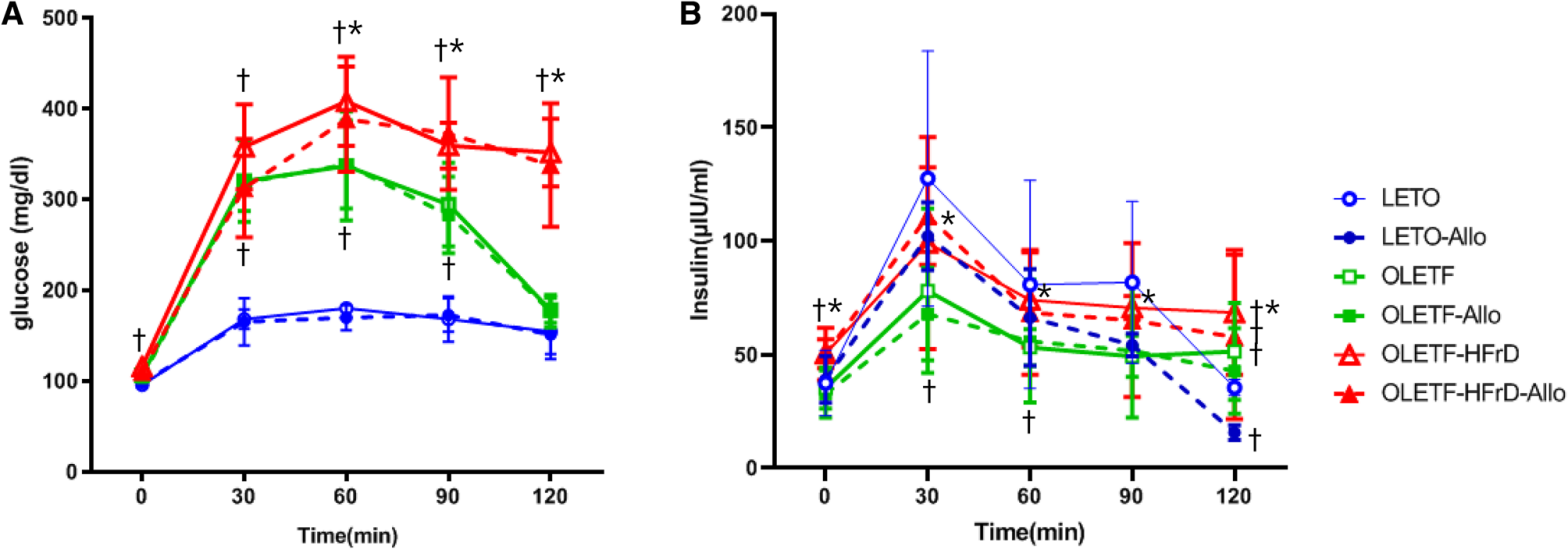
Effects of HFrD and allopurinol treatment on glucose tolerance test. (**A**) Glucose concentrations and (**B**) insulin concentrations in LETO, LETO-Allo, OLETF, OLETF-Allo, OLETF-HFrD, and OLETF-HFrD-Allo group during intraperitoneal glucose tolerance test (IPGTT). IPGTT were conducted. 2 g/kg glucose was intraperitoneally administered to rats after overnight fasting. And blood samples were collected for the measurement of biochemical parameters at 0 min, 30 min, 60 min, 90 min, and 120 min after glucose injection. Data are expressed as mean ± SE. †*P* < 0.05, versus the LETO group. *P < 0.05, versus OLETF group. LETO, Long-Evans Tohushima Otsuka; LETO-Allo, LETO rats treated with allopurinol; OLETF, Otsuka Long-Evans Tokushima Fatty rats; OLETF-Allo, OLETF rats treated with allopurinol; OLETF-HFrD, OLETF rats fed a high fructose diet; OLETF-HFrD-Allo rats, OLETF rats fed a high fructose diet treated with allopurinol.

According to the intraperitoneal glucose tolerance test (IPGTT) results, fasting glucose levels of the OLETF-HFrD and OLETF-HFrD-Allo groups were significantly higher than those of LETO rats (*P* < 0.05). The glucose levels at 30, 60, and 90 min and AUC (0–120) after intraperitoneal glucose loading in OLETF, OLETF-Allo, OLETF-HFrD and OLETF-HFrD-Allo groups were significantly higher than those of LETO rats (*P* < 0.05). Glucose levels at 60, 90, and 120 min and AUC (0–120) after intraperitoneal glucose loading in the OLETF-HFrD and OLETF-HFrD-Allo groups were significantly higher than those of OLETF rats (*P* < 0.05). However, the glucose levels of the allopurinol treatment groups were not significantly different from those of each control group. (LETO vs. LETO-Allo, OLETF vs. OLETF-Allo, OLETF-HFrD vs. OLETF-HFrD-Allo) (Fig. 1A and Table 2).

**Table 3:** Primer sequences and conditions for real-time PCR.

Target gene (Accession number)	Primers (forward, reverse)	Annealing Tm (°C)	Size (bp)
SREBP-1c (NM 001,276,707.1)	5′-CATCAACAACCAAGACAGTG-3′ 5′-GAAGCAGGAGAAGAGAAGC-3′	52	126
SCD-1 (NM 139,192.2)	5′-GGAACATCTGCCAGGGATCT-3′ 5′-AGTCGGGGAAGGTTCAACAC-3′	60	108
PPARα (NM 013,196.1)	5′-TACAGATGAGTCCCCTGGCA-3′ 5′-TCCAAAACGGATTGCATTGT-3′	52	256
CPT-1 (NM 031,559.2)	5′-GCATGATCGCAAAGATCAGT-3′ 5′-TGGTAGGAGAGCAGCACCTT-3′	55	156
TNF-α (X66539.1)	5′-AGATGGGCTGTACCTTATCTACTCC-3′ 5′-ACAGAGCAATGACTCCAAAGTAGA-3′	55	314
PAI-1 (NM 012,620.1)	5′-CCACGGTGAAGCAGGTGGACT-3′ 5′-TGCTGGCCTCTAAGAAGGGG-3′	55	195

Data are expressed as mean ± SE.

LETO, Long-Evans Tohushima Otsuka; LETO-Allo, LETO rats treated with allopurinol; OLETF, Otsuka Long-Evans Tokushima Fatty rats; OLETF-Allo, OLETF rats treated with allopurinol; OLEFT-HFrD, OLETF rats fed with high fructose diet; OLETF-HFrD-Allo rats, OLETF rats fed with high fructose diet which were treated with allopurinol; HFrD, high fructose-diet; AUC, Area under the curve; IGI 30, insulinogenic index 30 min (ΔInsulin30/ΔGlucose30); IGI 120, insulinogenic index 120 min (ΔInsulin120/ΔGlucose120); HOMA-beta, homeostatic model assessment of beta cell function; HOMA-IR, homeostatic model assessment of insulin resistance.

^†^*P* < 0.05 versus LETO group, *P < 0.05 versus OLETF group, #*P* < 0.05 versus OLETF-HFrD group.

Fasting insulin levels in the OLETF-HFrD and OLETF-HFrD-Allo groups were significantly higher than those in LETO rats (*P* < 0.05). The insulin levels at 30, 60, and 90 min, as well as AUC (0–120) after intraperitoneal glucose loading, IGI30, and HOMA-beta, in the OLETF and OLETF-Allo groups were significantly lower than those of LETO rats (*P* < 0.05). The insulin levels at 30, 60, 90, and 120 min, and AUC (0–120) after glucose loading, HOMA-beta, and HOMA-IR, of OLETF-HFrD rats were significantly higher than those of OLETF rats. The insulin levels at 120 min and AUC (0–120) of LETO-Allo were significantly decreased compared to those in the LETO group. However, those of OLETF-Allo and OLETF-HFrD-Allo numerically decreased and were not significantly lower than those of OLETF and OLETF-HFrD, respectively. However, the OLETF-HFrD-Allo group had significantly higher HOMA-beta levels and lower HOMA-IR levels than the OLETF-HFrD group (Fig. 1B and Table 2).

### Effects of HFrD and allopurinol treatment on hepatic steatosis

**Figure 2:**
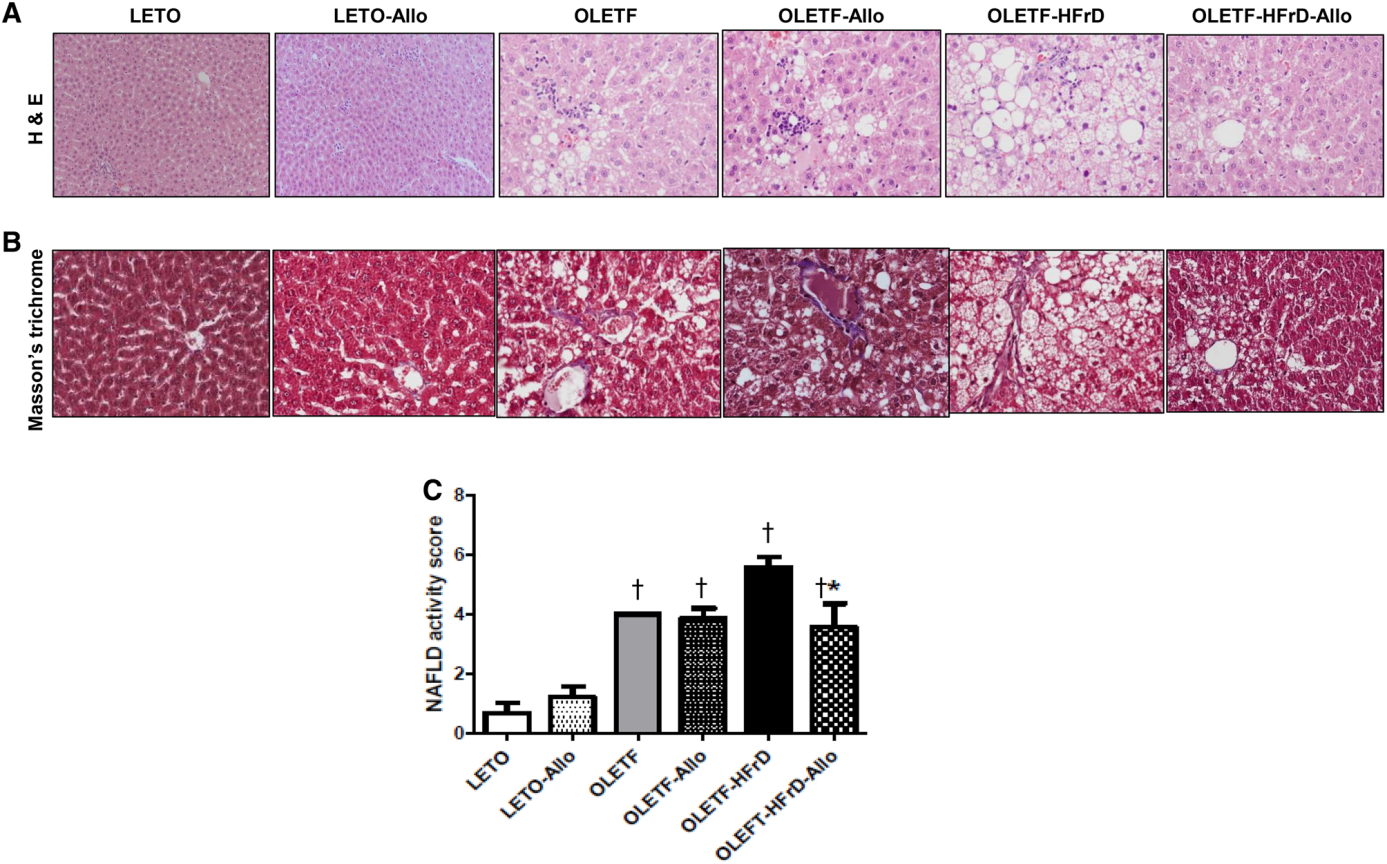
Liver pathology and NAFLD activity score after 16 weeks of LETO, OLETF, and HFrD-fed OLETF rats with or without allopurinol treatment. Liver morphology was visualized and evaluated using light microscopy (400 × magnification) on tissue sections subjected to hematoxylin and eosin (H&E) and Masson’s trichrome stains. (**A**). H&E-stained sections from rats in the OLETF and OLETF-HFrD group showed significant hepatic lipid accumulation compared to LETO group. Hepatic steatosis was decreased in the OLETF-HFrD-Allo group. (**B**). Significant fibrotic changes were not observed in all experimental groups. (**C**). NAFLD activity score was significantly elevated in the OLETF and OLETF-HFrD group compared to LETO group. Also, it was significantly decreased in OLETF-HFrD-Allo group, compared to OLETF-HFrD group. † *P* < 0.05 versus LETO group. *P < 0.05 versus OLETF-HFrD group. LETO, Long-Evans Tohushima Otsuka; LETO-Allo, LETO rats treated with allopurinol; OLETF, Otsuka Long-Evans Tokushima Fatty rats; OLETF-Allo, OLETF rats treated with allopurinol; OLETF-HFrD, OLETF rats fed with high fructose diet; OLETF-HFrD-Allo rats, OLETF rats fed with high fructose diet which were treated with allopurinol.

Histological analysis of liver tissue showed pronounced accumulation of lipid droplets in OLETF, OLETF-Allo, OLETF-HFrD, and OLETF-HFrD-Allo group rats compared to LETO rats. However, allopurinol treatment ameliorated HFrD-induced fat accumulation in the OLETF-HFrD-Allo group compared to that in the OLETF-HFrD group (Fig. 2A). Hepatic fibrosis was evaluated using Masson’s trichrome staining and was not observed in any of the six groups (Fig. 2B). The NAFLD activity score (NAS) was significantly higher in the OLETF, OLETF-Allo, OLETF-HFrD, and OLETF-HFrD-Allo group rats than in LETO rats. The NAS was significantly decreased in the OLETF-HFrD-Allo group compared to that in the OLETF-HFrD group (*P* < 0.05, Fig. 2C).

### Effects of HFrD and allopurinol treatment on hepatic expressions of lipid metabolism genes

**Figure 3:**
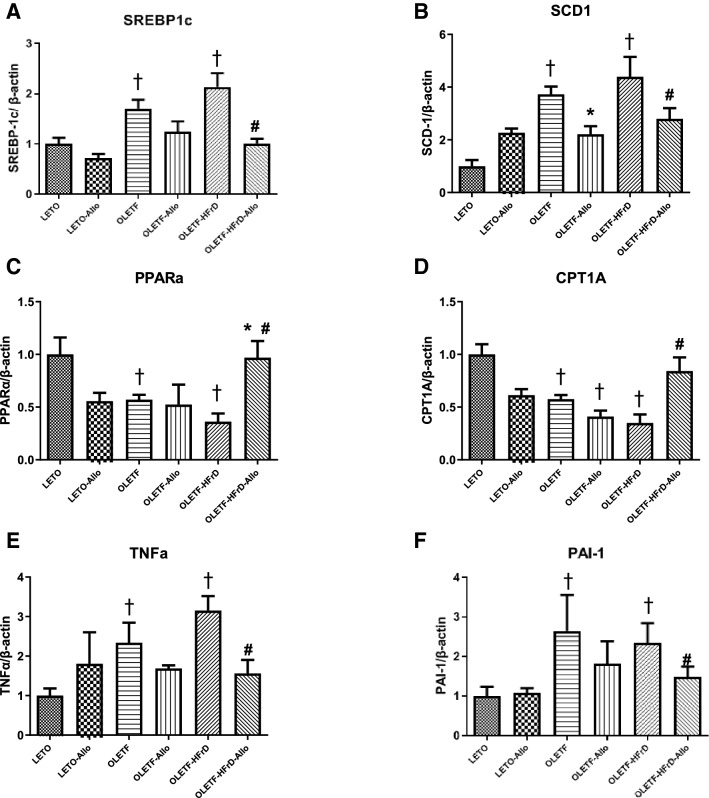
Hepatic mRNA expression of lipid metabolism genes and pro-inflammatory cytokine genes. Hepatic expression of genes was evaluated using real-time PCR. (**A** and **B**): Lipogenic genes expressions (SREBP-1c and SCD-1) were significantly upregulated in the OLETF and OLETF-HFrD groups compared to LETO group and significantly downregulated in the OLETF-HFrD-Allo group compared to the OLETF-HFrD group. (**C** and **D**): The expressions of lipid oxidation genes (PPARα and CPT-1) were significantly suppressed in the OLETF and OLETF-HFrD groups compared to the LETO group. However, allopurinol-treatment (OLETF-HFrD-Allo group) ameliorated the downregulation of lipid oxidation genes observed in OLETF-HFrD group. (**E** and **F**): Proinflammatory cytokine genes (TNF-α and PAI-1) significantly increased in the OLETF and OLETF-HFrD groups, and this increase was abolished in the OLETF-HFrD-Allo group. Data are expressed as mean ± SE. †*P* < 0.05 versus LETO group, *P < 0.05 versus OLETF group, #*P* < 0.05 versus OLETF-HFrD group LETO, Long-Evans Tohushima Otsuka; LETO-Allo, LETO rats treated with allopurinol; OLETF, Otsuka Long-Evans Tokushima Fatty rats; OLETF-Allo, OLETF rats treated with allopurinol; OLETF-HFrD, OLETF rats fed with high fructose diet; OLETF-HFrD-Allo rats, OLETF rats fed with high fructose diet which were treated with allopurinol; SREBP-1c, sterol regulatory element-binding protein 1c; SCD-1, stearoyl-CoA desaturase 1; PPARα, peroxisome proliferator-activated receptor-alpha; CPT-1, carnitine palmitoyltransferase 1; TNF-α, tumor necrosis factor-alpha; PAI-1, plasminogen activator inhibitor 1.

To determine whether HFrD and uric acid-lowering therapy affected the enzymes involved in hepatic lipid metabolism, hepatic expression of genes encoding lipogenic and fat oxidation enzymes was evaluated using real-time PCR. Expression of lipogenic genes, including sterol regulatory element-binding protein 1c (SREBP-1c) and stearoyl-CoA desaturase 1 (SCD-1) was significantly upregulated in the OLETF and OLETF-HFrD groups compared to LETO group. However, expressions of SREBP-1c and SCD-1 genes were significantly downregulated in the OLETF-HFrD-Allo group compared to the OLETF-HFrD group (*P* < 0.05, Fig. 3A, B). Peroxisome proliferator-activated receptor alpha (PPARα) and carnitine palmitoyltransferase 1 (CPT-1) were significantly downregulated in the OLETF and OLETF-HFrD groups compared to the LETO group. However, allopurinol treatment (OLETF-HFrD-Allo group) ameliorated the downregulation of lipid oxidation genes observed in the OLETF-HFrD group (*P* < 0.05, Fig. 3C,D). Therefore, the expression of the lipogenic genes of the OLETF-HFrD-Allo group was significantly suppressed compared to that in the OLETF-HFrD group. Additionally, the expression of lipid oxidation genes in the OLETF-HFrD-Allo group was significantly upregulated compared to that in the OLETF-HFrD group.

### Effects of HFrD and allopurinol treatment on hepatic expression of pro-inflammatory cytokine genes

The gene expression levels of hepatic pro-inflammatory cytokines were evaluated to determine the effect of allopurinol treatment on liver inflammation. Hepatic mRNA expression of tumor necrosis factor-alpha (TNF-α) was significantly increased in the OLETF and OLETF-HFrD groups, and this increase was abolished in the OLETF-HFrD-Allo group (*P* < 0.05, Fig. 3E). Similarly, mRNA expression of plasminogen activator inhibitor 1 (PAI-1) was significantly elevated in the OLETF and OLETF-HFrD groups, but this increase was abolished by allopurinol treatment (OLETF-HFrD-Allo group) (*P* < 0.05, Fig. 3F).

### Effects of HFrD and allopurinol treatment on the hepatic ER stress pathway

**Figure 4:**
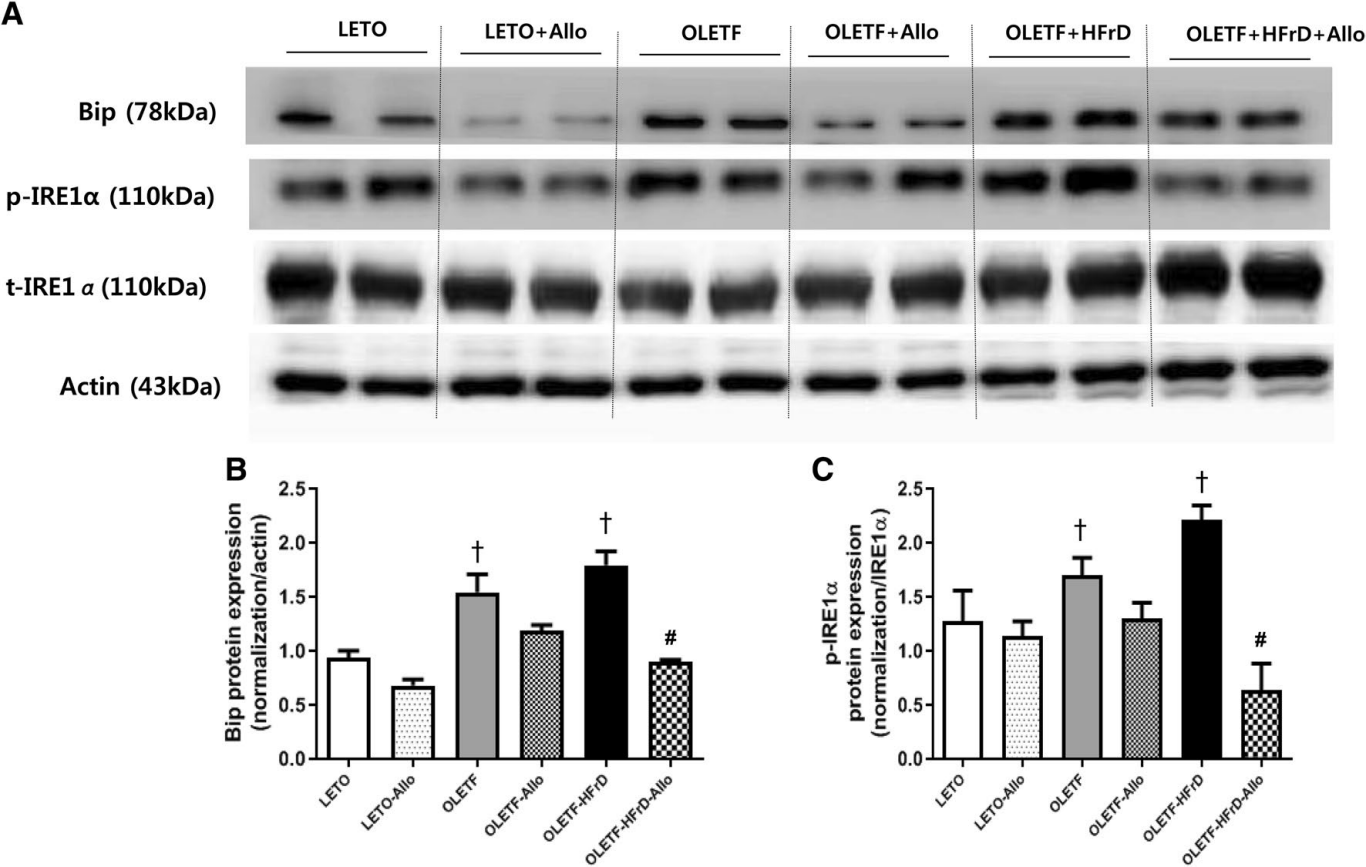
Hepatic ER stress pathway. BiP, p-IRE1α, and t-IRE1α protein expression levels were determined by Western blot analysis. Β-actin was used as a loading control. (**A**) OLETF and OLETF-HFrD groups significantly increased BiP over-expression and phosphorylation of IRE1 compared to LETO group. allopurinol-treatment significantly attenuated the HFrD-induced increased expression of BiP, and p-IRE1 in the OLETF-HFrD-Allo group compared to OLETF-HFrD group. (**B**,**C**). Relative protein expression was quantified using densitometry. *P < 0.05 versus NC group; #*P* < 0.05 versus HFrD group. ‘Full-length blots/gels’ are presented in Supplementary Fig. 1. LETO, Long-Evans Tohushima Otsuka; LETO-Allo, LETO rats treated with allopurinol; OLETF, Otsuka Long-Evans Tokushima Fatty rats; OLETF-Allo, OLETF rats treated with allopurinol; OLETF-HFrD, OLETF rats fed with high fructose diet; OLETF-HFrD-Allo rats, OLETF rats fed with high fructose diet which were treated with allopurinol; BiP, immunoglobulin heavy chain-binding protein; p- and t-IRE1, phosphorylated, and total inositol-requiring enzyme 1.

To examine the effect of fructose and allopurinol on the hepatic endoplasmic reticulum (ER) stress pathway activation, BiP, p-IRE1α, and t-IRE1α protein expression levels were determined by Western blot analysis (Fig. 4). Β-actin was used as a loading control. The expression of BiP and p-IRE1α in OLETF and OLETF-HFrD groups significantly increased (*P* < 0.05) compared to LETO group (Fig. 4). Allopurinol treatment tended to decrease the expression of Bip and p-IRE1α in the LETO-Allo and OLETF-Allo group compared to LETO and OLETF group, respectively. However, allopurinol-treatment significantly attenuated the HFrD-induced increased expression of BiP and p-IRE1 in the OLETF-HFrD-Allo group compared to the OLETF-HFrD group (*P* < 0.05) (Fig. 4).

## Discussion

The present study showed that HFrD aggravated the development of hepatic steatosis, obesity, hyperinsulinemia, glucose intolerance, dyslipidemia, and hyperuricemia in OLETF rats with a phenotype of metabolic syndrome compared to LETO rats. These effects, specifically, fatty liver, dyslipidemia, hyperuricemia, and insulin resistance, were significantly ameliorated by the administration of allopurinol (a uric acid-lowering agent) in the HFrD-fed OLETF rats but not in the OLETF rats. Also, allopurinol significantly downregulated expression of lipogenic genes, upregulated lipid oxidation genes, downregulated hepatic pro-inflammatory cytokine genes, and decreased ER-stress induced protein expressions compared with those of OLETF-HFrD group. Therefore, it is suggested that uric acid plays an important role in the development of high fructose-induced hepatic steatosis.

NAFLD is a one of the clinical manifestations of metabolic syndrome, which consists of insulin resistance, obesity, hyperglycemia, hypertension, and dyslipidemia^2^. Recently, metabolic dysfunction-associated fatty liver disease (MAFLD) became the updated single overarching nomenclature to describe the disease^22^. MAFLD is commonly observed in clinical practice in association with overweight/obesity^22^. Some studies have shown that allopurinol does not affect weight change in fructose-fed animals^23^. The present study showed that fructose diet-induced weight gain and increase in epididymal fat in the OLETF-HFrD group were significantly reduced in the allopurinol-treated OLETF-HFrD-Allo group. We did not assess the amount of dietary intake or metabolic rate. However, Nakagawa et al.^24^ stated that allopurinol prevented weight gain in HFrD-fed rats, although allopurinol did not have any specific effects on food intake. The mechanism of the weight loss effect of allopurinol should be studied further. In addition, a decrease in epididymal fat weight and increased expression of hepatic lipid oxidation genes suggest that studies on adipose tissue are needed.

There is an intimate association between MAFLD and T2DM: more than 70% of patients with T2DM have MAFLD. Moreover, HFrD induced hepatic steatosis by direct modulation of hepatic lipogenesis and indirect action through uric acid, a metabolite of fructose^7,8^. This study showed that OLETF-HFrD rats showed higher HOMA-IR, fasting insulin, HOMA-beta, and AUC (0–120) of insulin compared to OLETF rats. Decreased hepatic insulin clearance usually results in increased insulin secretion to compensate for insulin resistance. Chronic hyperinsulinemia stimulates hepatic de novo lipogenesis and hepatic steatosis. Therefore, impaired hepatic insulin clearance can be linked to hepatic insulin resistance and hepatic steatosis^25^. This suggests that HFrD may impair hepatic insulin clearance, which can also contribute to hepatic steatosis. Further studies, on the effect of HFrD on hepatic insulin clearance, are needed. The present study also showed that allopurinol treatment improves HFrD-induced insulin resistance. The HOMA-IR of OLETF-HFrD-Allo rats was significantly lower than that of OLETF-HFrD rats. Insulin AUC (0–120) also decreased numerically, despite an increase in the insulinogenic index (IGI) to 30. However, hyperglycemia did not improve after allopurinol treatment. This may be due to impaired pancreatic beta cell function in the OLETF rats. In our study, HOMA-beta, insulin 30 min, and AUC (0–120) of insulin after the glucose loading test were significantly lower in OLETF rats than in LETO rats. Thus, allopurinol did not improve hyperglycemia in the OLETF-Allo and OLETF-HFrD-Allo groups. Further studies on the effect of allopurinol on glucose metabolism, in a non-diabetic animal model, are needed. In addition, according to Andrikopoulos et al.^26^, OGTT was superior to IPGTT in testing glucose metabolism under conditions of insulin resistance. In our study, the IPGTT results did not show a significant difference in glucose metabolism. Therefore, the OGTT would be more appropriate to assess whether the reduction in steatosis improves insulin resistance, with respect to glucose metabolism.

Excessive fructose consumption causes NAFLD through various mechanisms. Fat accumulation in the liver occurs when the input of fatty acids (i.e., via uptake or de novo synthesis) exceeds output (i.e., via degradation or export)^14^. After fructose enters the liver, it is first converted by the glycolytic pathway to pyruvate, and then metabolized to acetyl-CoA in the hepatic mitochondrial tricarboxylic acid (TCA) cycle. An elevated fructose load leads to the generation of excess acetyl-CoA that exits the mitochondria into the cytosol where it converts to citrate, a substrate for de novo lipogenesis^14^. The present study showed that the hepatic mRNA expression of SREBP-1c and SCD-1 were significantly increased in OLETF and OLETF-HFrD rats compared to LETO rats. In addition, there was no significant difference in lipogenic gene expression between OLETF and OLETF-HFrD rats. However, other studies have indicated that fructose can upregulate the expression of genes involved in de novo lipogenesis by stimulating the activation of transcription factors, including carbohydrate response element-binding protein (ChREBP) and SREBP-1c^27^. This discrepancy may be caused by the use of different animal strains and/or species in studies. Previous studies have generally used non-obese and non-diabetic rats, while this study used OLETF rats, an animal model of metabolic syndrome. Lipogenic gene expression is usually elevated in OLETF rats compared with control LETO rats, even in the absence of any specific dietary manipulation^28^. In the current study, allopurinol treatment significantly downregulated SREBP1C and SCD1 in the OLETF-HFrD-Allo group compared to the OLETF-HFrD group. This suggests that allopurinol treatment could suppress HFrD-induced lipogenesis.

There is a close relationship between lipogenesis and fatty acid oxidation processes in the liver. De novo lipogenesis increases the malonyl-CoA level, which inhibits CPT-1 enzyme mediation of the entry of long-chain acyl-CoA coenzymes into the mitochondria and the initiation of fatty acid breakdown via beta-oxidation^29^. PPARα, part of the superfamily of ligand-activated nuclear hormone receptors, is also associated with hepatic lipid metabolism, as it regulates mitochondrial beta-oxidation enzymes^30^. The present study showed that OLETF rats and OLETF-HFrD rats had significantly downregulated PPARα and CPT-1 expressions, leading to fatty liver pathogenesis. These results were consistent with other studies that showed the reduction of hepatic PPARα expression by excessive fructose in rats, resulting in the inhibition of fatty acid oxidation enzymes and suppression of hepatic lipid export^31^. Furthermore, the present study showed that allopurinol-treatment ameliorated HFrD-induced hepatic steatosis in OLETF rats through the downregulation of lipogenic gene expression and upregulation of lipid oxidation genes expression. Indeed, drugs that can increase PPARα activity, such as fenofibrate or statins, have been shown to prevent NAFLD development in vivo^32^. As such, allopurinol could improve fatty liver through the modulation of PPARα signaling, and its efficacy serves as evidence that uric acid is directly involved in the development of NAFLD. In this study, we measured only mRNA expression related to lipogenesis and lipid oxidation but did not measure protein expression. The efficiency of translation or regulation of translation might affect the final protein content of specific proteins, depending on many variables^33^. Further studies are required to understand protein expression related to lipid metabolism.

Lipotoxicity due to excessive accumulation of lipids in hepatocytes can lead to inflammation, fibrosis, and pathological angiogenesis^34^. Inflammatory responses are caused by oxidative stress, reactive oxygen species, and activation of cytokines such as TNF-α^2,14^. Previous studies have indicated that hepatic TNF-α expression is significantly increased in mice fed with an HFrD^35^ and TNF-α has a critical role in the pathogenesis of NAFLD^35,36^. PAI-1 is an acute-phase protein, and in vitro and in vivo studies have shown that its hepatic expression is regulated by TNF-α^37^. Plasma PAI-1 levels are associated with hepatic steatosis severity in obese humans and genetically obese mice^38^. Accordingly, PAI^-/-^ mice are protected from lipid accumulation after alcohol administration^37^. This study found that HFrD increased the hepatic expression of TNF-α and PAI-1. This was consistent with a recent study that reported increased hepatic PAI-1 expression with fructose administration and the important role PAI-1 plays in fructose-mediated liver injury^39^. The present study also showed that allopurinol treatment significantly reduced the hepatic gene expression of pro-inflammatory cytokines. This phenomenon may occur via inhibition of xanthine oxidase, which has been shown to reduce liver injury by decreasing reactive oxygen species formation^40^ and modulating transcription factors that regulate the production of pro-inflammatory cytokines^41^.

Disruption of ER homeostasis can lead to hepatic steatosis, inflammation, and insulin resistance^42^. The ER is an intracellular organelle that plays an important role in protein synthesis, folding, modification, and trafficking. When the protein folding or modification processes are disrupted, ER stress is induced, and the unfolded protein response (UPR) is activated^43^. There are three main branches of the UPR: the protein kinase RNA-like ER kinase (PERK) pathway, the activated transcription factor 6 (ATF6) pathway, and the IRE1 pathway. BiP also referred to as glucose-regulated protein 78, is an intra-luminal chaperone protein that plays a key role in initiating the UPR. Under normal conditions, BiP binds to UPR transducers such as PERK, ATF, and IRE1, and acts to suppress pathway activation. When ER stress is induced, BiP is separated from UPR transducers, facilitating the initiation of signaling transduction in each pathway. The present study found that as BiP expression and IRE1-XBP-1 pathway activation increased, fructose consumption induced hepatic ER stress pathway activation. Previous research showed that fructose-treatment induced fat accumulation in primary hepatocytes via induction of ER stress^44^ and in vivo^45^. Uric acid has been demonstrated to increase ER stress in various cell types, including primary hepatocytes^27^, glomerular mesangial cells^46^, and endothelial cells^47^. Of the three UFR pathways, the IRE1-XBP-1 pathway has been reported as a regulator of hepatic metabolism. XBP-1 increases hepatic expression of lipogenic genes such as SCD-1^48^, and the activation of c-Jun N-terminal kinase by IRE1α induces hepatic insulin resistance^49^. Additionally, the IRE1α-XBP-1 pathway plays a key role in the regulation of hepatic very-low-density lipoprotein assembly and secretion^50^. Finally, a previous study showed that the hepatic-specific deletion of IRE1α results in the development of severe hepatic steatosis when ER stress is induced^51^. Therefore, HFrD-induced activation of ER stress mechanisms may contribute to fatty liver development.

Recently, many in vitro studies using hepatocytes have evaluated the direct effects of uric acid on hepatic steatosis. Lanaspa et al. indicated that uric acid can stimulate fat accumulation via the generation of mitochondrial oxidative stress in HepG2 cells and is related to the mechanisms by which fructose induces fatty liver^8^. Furthermore, they also reported that uric acid can upregulate fructokinase, resulting in increased lipid accumulation via sensitizing hepatocytes to fructose metabolism^8^. Another study has revealed that uric acid induces fat accumulation in hepatocytes by ER stress-mediated SREBP-1c activation^27^. Another study has also shown that uric acid induces hepatic steatosis and insulin resistance in vitro and in vivo through the activation of the NOD-like receptor family, pyrin domain-containing protein 3 (NLRP3) inflammasome^52^. These studies support the hypothesis that uric acid generation from fructose metabolism accelerates fructose-induced NAFLD. The present study is, to the best of my knowledge, the first in vivo study to show that allopurinol inhibited fructose load-induced activation of ER stress pathways, especially the IRE1 related pathway. Therefore, it is suggested that uric acid may be involved in the fructose-induced ER stress pathway activation.

Allopurinol is a xanthine oxidase inhibitor, which has been widely used for the treatment of gout. In rat studies that evaluated the effects of uric acid-lowering therapy on metabolic syndrome, allopurinol was used at a dose range of 5 mg/kg to 30 mg/kg^20,53,54^. The present study used 10 mg/kg/day of allopurinol that was found to be effective in significantly reducing uric acid levels and had been previously recommended to minimize organ toxicity^55^. Considering FDA guidance for estimating a human efficient dose, an allopurinol dose of 10 mg/kg/day in rats correspond to 100–300 mg/day in humans, per standard doses used the treatment of hyperuricemia in patients^56^. Therefore, the dosage used in the present study and the results generated with that dosage may be useful for a future study that evaluates the efficacy of allopurinol treatment in human patients with NAFLD.

Limitations in the present study include the following: First, allopurinol treatment did not improve hyperglycemia despite significant amelioration of hepatic steatosis and insulin resistance index such as HOMA-IR and AUC of insulin. Data were not collected from muscle or adipose tissues which are peripheral tissues that may have contributed to insulin resistance and the pancreas, which are sources of insulin secretion. Further studies are also required to evaluate alterations in the expression of insulin resistance-related genes in muscle or adipose tissues and insulin secretion-related genes in pancreatic tissues. Second, allopurinol treatment reduced HFrD-induced weight gain and epididymal fat weight. We did not measure dietary parameters or metabolic rate and did not estimate the weight loss effect of allopurinol. Therefore, further studies are needed to evaluate the mechanism of the weight loss effect of allopurinol in HFrD-induced obesity animal models. Third, in the present study, allopurinol decreased serum UA levels and improved NAFLD by decreasing de novo lipogenesis, fatty acid oxidation, and ER stress; however, we do not know whether reduction of serum UA level directly influences the improvement of NAFLD. Usually, serum uric acid levels increase in patients with metabolic syndrome due to decreased clearance of uric acid. Hyperuricemia itself may aggravate the progression of NAFLD, which can be partially protected by allopurinol through amelioration of hyperuricemia-induced NAFLD pathogenesis. However, this hypothesis should be evaluated in other in vivo models of hepatic steatosis and in vitro experiments, to examine whether high fructose with or without uric acid influences the progression of NAFLD, even though the in vitro UA treatment is not the same as the UA concentration as a metabolite of fructose.

In conclusion, HFrD induces the development of significant hepatic steatosis, obesity, hyperinsulinemia, glucose intolerance, dyslipidemia, and hyperuricemia. In addition, allopurinol, uric acid lowering agent treatment reduces serum uric acid and ameliorates HFrD-induced hepatic steatosis through the modulation of hepatic lipid metabolism, expression of pro-inflammatory cytokines, and the ER stress pathway, independent of glucose metabolism. Therefore, uric acid may have a direct role in the development of fructose-induced hepatic steatosis, and uric acid lowering therapy could be a candidate for the prevention or treatment of NAFLD.

## Methods

### Ethics statement

All animal experiments were approved by the Institutional Animal Care and Use Committee of Kyung Hee University Hospital at Gangdong (KHNMC-IACUC-2016–04). Principles of laboratory animal care (NIH publication no. 85–23, revised 1996; http://grants1.nih.gov/grants/olaw/references/phspol.htm) were followed, as well as specific national laws when applicable. All animal facilities were approved by the Use Committee of the Kyung Hee University Hospital at Gangdong. The use of animals was kept to an absolute minimum, as per requirement, to achieve statistical significance for validation. This study was conducted in compliance with the Animal Research: Reporting In Vivo Experiments (ARRIVE) guidelines.

### Animal

Otsuka Long-Evans Tokushima Fatty (OLETF) male rats, which are characterized as insulin resistant, obese, hyperglycemic, and dyslipidemia, were used^20^. Male LETO rats were used as controls. LETO and OLETF male rats were purchased from Tokushima Research Institute (Otsuka Pharmaceutical, Tokushima, Japan) and maintained on a 12 h light/dark cycle at 22 °C to 26 °C with ad libitum access to food and water.

### Experimental design

The 30-week-old LETO and OLETF male rats were randomly divided into six groups of 10 rats as follows: (1) LETO rats fed a normal chow diet (LETO group), (2) LETO rats treated with allopurinol (LETO-Allo group), (3) OLETF rats fed a normal chow diet (OLETF group), (4) OLETF rats treated with allopurinol (OLETF-Allo group), (5) HFrD-fed OLETF rats (OLETF-HFrD group), and (6) HFrD-fed OLETF rats with allopurinol treatment (OLETF-HFrD-Allo group). The HFrD contained 60% fructose (Central Lab Animal Inc., Seoul, Republic of Korea). Allopurinol (Sigma-Aldrich, St. Louis, MO, USA), a xanthine oxidase inhibitor, was used to block the generation of intracellular uric acid and was administered through drinking water at a dose of 10 mg/kg/day. Each intervention was continued for 16 weeks. At the end of the study, body weights were measured, and IPGTTs were conducted. Glucose (2 g/kg) was intraperitoneally administered to rats after overnight fasting. Blood samples were collected for the measurement of biochemical parameters at 0, 30, 60, 90, and 120 min after glucose injection.

### Termination of the experiment

After the IPGTT study at the end of the experimental period (16 weeks), rats were sacrificed by an overdose of diethyl ether^57^. Blood was collected and serum was stored at − 20 °C until used for further analysis. Liver and pancreas were removed and weighed to calculate relative organ weight. A small portion of liver was excised and fixed in in 4% formalin for histopathological examination. The remaining parts of liver were stored at − 70 °C until used for RNA and protein analysis^58^.

### Measurement of serum biochemical parameters

Serum fasting glucose, total cholesterol, triglyceride (TG), aspartate aminotransferase (AST), and alanine aminotransferase (ALT) concentrations were measured using a commercial kit (Asan Pharm. Co., Ltd, Hwaseong-si, Gyeonggi-do, Republic of Korea). Serum fasting insulin concentrations were determined using a rat insulin ELISA kit (EZRMI-13 K, Millipore, Billerica, MA, USA), and uric acid concentrations were measured using a colorimetric assay kit (Abcam, Cambridge, MA, USA). Hemoglobin A1c (HbA1c) concentrations were determined using an automatic analyzer (DCA 2000 System, Bayer, Elkhart, IN, USA).

The IGI, an indicator of beta-cell function, was defined as the ratio of the change in insulin concentration (Δ Insulin) to the change in glucose concentration (Δ Glucose)^59^. IGI at 30 min and AUC (0–120) of insulin and glucose after glucose administration were calculated from the IPGTT data as follows:

IGI (Δ Insulin 30/Δ Glucose 30) = (insulin at 30 min – insulin at 0 min [μIU/mL])/(glucose at 30 min – glucose at 0 min [mg/dL]). AUC (0–120) = Area under the curve from 0 to 120 min for glucose and insulin each.

The homeostasis model assessment of beta-cell function (HOMA-beta) and insulin resistance (HOMA-IR)^60^ were calculated using fasting glucose and insulin concentrations as follows:

HOMA-beta = 360 × fasting insulin (μIU/mL) / (fasting glucose [mg/dL] – 63); HOMA-IR = fasting glucose (mg/dL) × fasting insulin (μIU/mL)/405.

### Histological analysis of liver tissue

The paraffin-embedded sections were stained with hematoxylin and eosin (H&E) or Masson’s trichrome. A single pathologist, who was blinded to the experimental details, using light microscopy (400 × magnification), performed liver histological analysis. The NAFLD activity score (NAS) was calculated using the Nonalcoholic Steatohepatitis Clinical Research Network (NASH CRN) scoring system^61^. This scoring system analyzes four components to evaluate histological changes of NAFLD, and each component is scored, based on the degree of morphological change, as follows: steatosis grade (0–3), lobular inflammation (0–3), hepatocellular ballooning (0–2), and fibrosis score (0, 1a, 1b, 1c, 2, 3, 4). The NAS was calculated as the sum of the scores for steatosis, lobular inflammation, and hepatocellular ballooning.

### Quantitative real-time PCR

Total hepatic RNA was extracted using TRIzol reagent (Invitrogen Corp., Carlsbad, CA, USA) according to the manufacturer’s instructions. Total RNA (1 μg) was reverse transcribed using AMV reverse transcriptase (TaKaRa Bio., Kyoto, Japan). Real-time polymerase chain reactions (PCR) were conducted using the Power SYBR Green PCR master mix (Applied Biosystems Inc., Foster City, CA, USA) in the StepOne Plus real-time PCR system (Applied Biosystems). Bioneer (Daejeon, Republic of Korea) designed the PCR primers. Primer sequences and PCR conditions are shown in Table 3. Each mRNA level was normalized to the β-actin mRNA level.

### Western blot analysis

Liver tissues were lysed in radioimmunoprecipitation assay (RIPA) lysis buffer (iNtRON Biotechnology, Seongnam-si, Gyeonggi-do, Republic of Korea). Samples were centrifuged at 13,000 rpm × 20 min at 4 ºC. Protein concentrations were quantified by the Bradford method. Proteins were loaded onto a 10% acrylamide gel, separated using SDS-PAGE, and transferred onto polyvinylidene difluoride membranes (Millipore). The membranes were incubated overnight at 4 °C in Tris-buffered saline-Tween 20 (TBST) with primary antibodies (1:1000 dilution) against the following: immunoglobulin heavy chain-binding protein (BiP, Cell Signaling Biotechnology, Beverly, MA, USA), phosphorylated inositol-requiring enzyme 1 (p-IRE1, Abcam), total IRE1 (t-IRE1, Abcam), x-box binding protein 1 (XBP-1, Abcam), and β-actin (Santa Cruz Biotechnology, Santa Cruz, CA, USA). After washing, the membranes were incubated with horseradish peroxidase-conjugated goat secondary antibodies (anti-rabbit or mouse antibodies, 1:5000 dilution, Thermo Scientific, Rockford, IL, USA) at room temperature for 1 h. Immunoreactive bands were enhanced and detected by chemiluminescence (ECL, Millipore), and band densities were quantified using ImageJ software (National Institutes of Health, Bethesda, MD, USA). To simultaneously investigate the expression of several proteins of different sizes on one membrane, the membrane was cut into sections and identified by attaching different antibodies. Therefore, we provided a cropped image rather than a full-length western blot image.

### Statistical analysis

Data were expressed as either mean ± SE or median with interquartile range. Each experiment was repeated in triplicate. Statistical analysis was performed using SPSS 18.0 software (Chicago, IL, USA). One-way analysis of variance was used followed by a post-hoc test with the Bonferroni procedure when data were normally distributed. Data not normally distributed were evaluated using the Kruskal–Wallis test; significant differences for multiple comparisons between groups were determined using the Mann–Whitney U-test with adjustment of the significance level for the decision criteria using a Bonferroni type adjustment. Statistical significance was defined as *P* < 0.05.

## Supplementary Information

Supplementary Information 1.
