# Supplementary material for: Allopurinol ameliorates high fructose diet induced hepatic steatosis in diabetic rats through modulation of lipid metabolism, inflammation, and ER stress pathway
Source: Sci Rep. 2021 May 10;11:9894. doi: 10.1038/s41598-021-88872-7 (PMC8110790; doi:10.1038/s41598-021-88872-7)
Supplement: Supplementary file 1 — Supplementary Information 1. [file 41598_2021_88872_MOESM1_ESM.pdf]

Allopurinol ameliorates high fructose diet induced hepatic steatosis in diabetic rats through modulation of lipid metabolism, inflammation, and ER stress pathway

Running title: The effect of allopurinol on hepatic steatosis

In-Jin Cho[a], Da-Hee Oh[a], Jin Yoo[a], You-Cheol Hwang[a,b], Kyu Jeung Ahn[a,b], Ho-Yeon Chung[a,b], Soung Won Jeong[c], Ju-Young Moon[d], Sang-Ho Lee[d], Sung-Jig Lim[e], In-Kyung Jeong[a,b]

[a]Department of Endocrinology and Metabolism, Kyung Hee University Hospital at Gangdong, Seoul, Korea

[b]Division of Endocrinology and Metabolism, Department of Internal Medicine, Kyung Hee University School of Medicine, Seoul, Korea

[c]Division of Gastroenterology and Hepatology, Department of Internal Medicine, Soonchunhyang University College of Medicine, Seoul, Korea

[d]Division of Nephrology, Department of Internal Medicine, Kyung Hee University School of Medicine, Seoul, Korea

[e]Department of pathology, Kyung Hee University School of Medicine, Seoul, Korea

Supplementary figure 1. Full-length blots/gels' of Fig 4

A) Bip

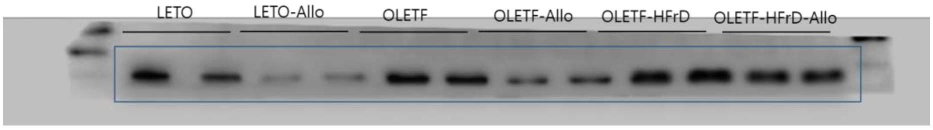

B) p-IRE1

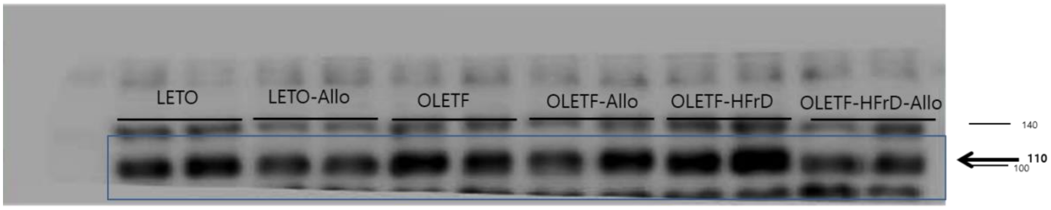

C) t-IRE1

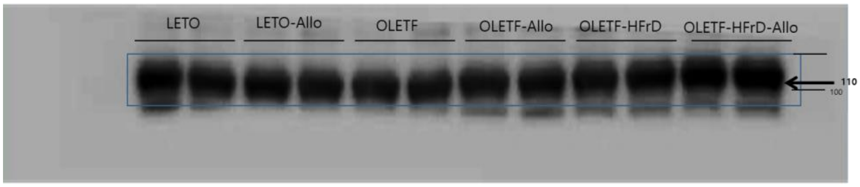

D) Actin

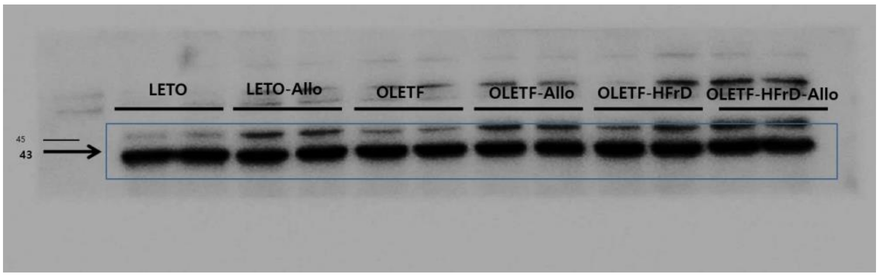

Supplementary figure 1. Full-length blots/gels' of Fig 4.

A) BiP expression, B) p-IRE1, C) t-IRE1, D) Actin of western blot data.

M is marker, NC, normal chow-diet fed OLETF rats; HFrD, high fructose-diet fed OLETF rats; HFrDAL, high fructose-diet fed OLETF rats with allopurinol treatment.

To simultaneously investigate the expression of several proteins of different sizes on one membrane, the membrane was cut into sections and identified by attaching different antibodies. Therefore, we provided a cropped image rather than a full-length image of western blot membrane.
